# 925. Oseltamivir usage in community settings: data from a household transmission study

**DOI:** 10.1093/ofid/ofad500.970

**Published:** 2023-11-27

**Authors:** Alexandra Mellis, Jessica E Biddle, James W Antoon, Son H McLaren, Edward Belongia, Yvonne A Maldonado, Natalie M Bowman, Katherine Ellingson, Suchitra Rao, Sheroi Johnson, Melissa Stockwell, Ellen Sano, Raul A Silverio, Ayla Bullock, Amy Yang, Quenla Haehnel, Huong McLean, Joshua Petrie, Clea Sarnquist, Prasanthi Govindaranjan, Sara H Goodman, Karen Lutrick, Karla I Ledezma, Kathleen Pryor, Edwin J Asturias, Yuwei Zhu, Jonathan Schmitz, Kimberly W Hart, Carlos G Grijalva, H Keipp Talbot, Melissa A Rolfes

**Affiliations:** Centers for Disease Control and Prevention, Atlanta, GA; Centers for Disease Control and Prevention, Atlanta, GA; Vanderbilt University Medical Center, Nashville, Tennessee; Columbia University Irving Medical Center, New York City, New York; Marshfield Clinic Research Institute, Marshfield, WI; Stanford University, Stanford, California; University of North Carolina, Chapel Hill, North Carolina; University of Arizona, Tucson, Arizona; University of Colorado School of Medicine, Aurora, Colorado; Centers for Disease Control and Prevention, Atlanta, GA; Columbia University Irving Medical Center, New York City, New York; Columbia University Irving Medical Center, New York City, New York; Columbia University Medical Center, New York, New York; University of North Carolina, Chapel Hill, North Carolina; University of North Carolina, Chapel Hill, North Carolina; University of North Carolina, Chapel Hill, North Carolina; Marshfield Clinic Research Institute, Marshfield, WI; Marshfield Clinic Research Institute, Marshfield, WI; School of Medicine, Stanford University, Palo Alto, California; Stanford University School of Medicine, Stanford, California; Stanford University School of Medicine, Stanford, California; University of Arizona College of Medicine, Tucson, Arizona; University of Arizona College of Medicine, Tucson, Arizona; University of Arizona College of Medicine, Tucson, Arizona; University of Colorado School of Medicine, Aurora, Colorado; Vanderbilt University, Nashville, Tennessee; Vanderbilt University Medical Center, Nashville, Tennessee; Vanderbilt University Medical Center, Nashville, Tennessee; Vanderbilt University Medical Center, Nashville, Tennessee; Vanderbilt University Medical Center, Nashville, Tennessee; Centers for Disease Control and Prevention, Atlanta, GA

## Abstract

**Background:**

Oseltamivir is the most common antiviral prescribed to treat influenza. There are limited data about oseltamivir receipt in uncomplicated influenza, including frequency of early discontinuation of oseltamivir.

**Methods:**

Individuals who tested positive for influenza were identified from outpatient clinics, emergency departments, or surveillance testing at seven sites in the United States from December 2021–March 2023. Index cases and their household contacts (HHC) enrolled ≤6 days after the first illness in the household, completed symptom/medication logs, and collected nasal swabs for influenza testing daily for 10 days. Oseltamivir receipt was classified by daily logs (ever/never receipt, and duration of use: early discontinuation [1–2 days], 3–4 days, or ≥5 days) among eligible persons (Methods 1). Addresses linked to the 2020 Social Vulnerability Index by census tract. Odds of oseltamivir receipt were estimated using binomial regression with household clustering.

Methods upload 1: Analytic flow diagram for assessment of use and discontinuation of oseltamivir in household settings, United States 2021-22 and 2022-23 influenza seasons.

**Figure:**
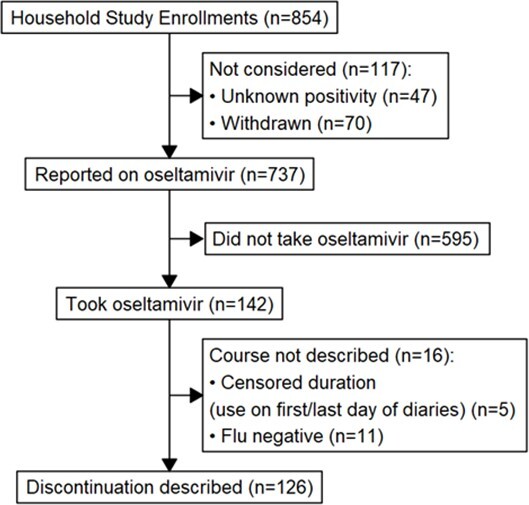

Discontinuation was defined as use for 1-2 days, compared to use for 3-4 or ≥5 days. If the participant reported oseltamivir on the first or last day of follow-up and did not report use for at least 3 days, the duration of oseltamivir usage was considered “censored” and discontinuation was not described.

**Results:**

Of 737 household members, 142 individuals (90/235 index cases, 40/284 infected HHC, 12/218 uninfected HHC) reported oseltamivir. Oseltamivir receipt was more common among those recruited in 2022–23 vs. 2021–22 and by participants who self-reported pre-existing conditions vs. those who did not. Oseltamivir receipt was less common among children 5–11 vs. adults 18–49 years (Results 1). Individuals from the most vulnerable census tracts were least likely to receive oseltamivir (Results 2). Among symptomatic infected persons, oseltamivir was typically initiated within 2 days of symptoms (76%). Of 126 individuals whose duration of oseltamivir was not censored by the start or end of follow-up, 19% reported receipt on only 1-2 days, 17% for 3-4 days, and 63% for ≥5 days. Compared to those who reported ≥5 days of oseltamivir receipt, people who took oseltamivir for 1–2 days had similar reported symptoms, including gastrointestinal symptoms, on the first day of illness and first day of oseltamivir (Results 3).

Results upload 1: Characteristics of individuals who did and did not report use of oseltamivir among individuals in households with a known influenza case, United States, 2021-22 and 2023-24 influenza seasons.

**Figure:**
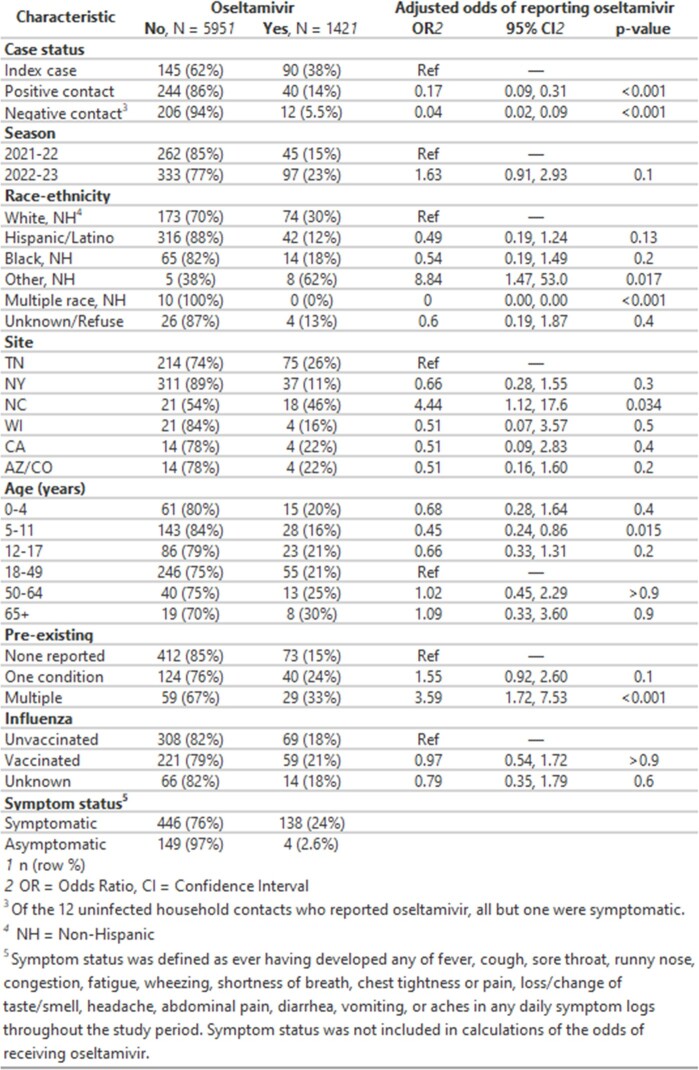

Results Upload 2. Social vulnerability of individuals who did and did not report use of oseltamivir among individuals in geocoded households with a known influenza case, United States, 2021-22 and 2023-24 influenza seasons.

**Figure:**
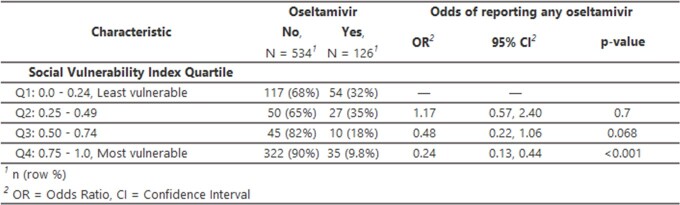

**Conclusion:**

Over a third of index cases received oseltamivir, and receipt differed by age and social vulnerability status. In this analysis, early discontinuation was not associated with initial symptom burden or symptoms, including gastrointestinal side-effects, after initiating oseltamivir.

**Figure:**
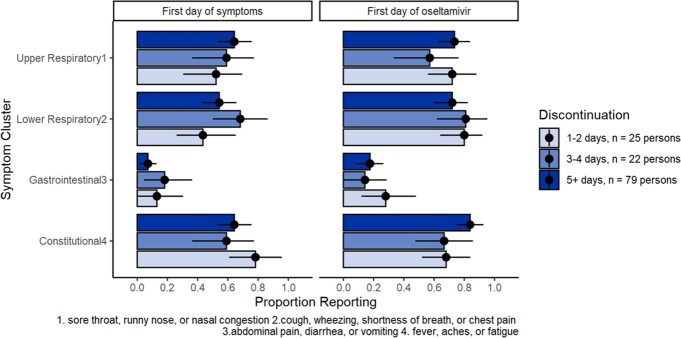

**Disclosures:**

**Edward Belongia, MD**, Seqirus: Grant/Research Support **Yvonne A. Maldonado, MD**, Pfizer: Grant/Research Support|Pfizer: Site Investigator, DSMB member **Suchitra Rao, MBBS, MSCS**, Sequiris: Advisor/Consultant **Huong McLean, PhD, MPH**, Seqirus: Grant/Research Support **Joshua Petrie, PhD**, CSL Seqirus: Grant/Research Support **Edwin J. Asturias, MD**, Hillevax: Advisor/Consultant|Moderna: Advisor/Consultant|Pfizer: Grant/Research Support **Carlos G. Grijalva, MD, MPH**, AHRQ: Grant/Research Support|CDC: Grant/Research Support|FDA: Grant/Research Support|Merck: Advisor/Consultant|NIH: Grant/Research Support|Syneos Health: Grant/Research Support

